# Effects of active vitamin D analogues on muscle strength and falls in elderly people: an updated meta-analysis

**DOI:** 10.3389/fendo.2024.1327623

**Published:** 2024-02-01

**Authors:** An Xiong, Haibo Li, Miaoying Lin, Feng Xu, Xuedi Xia, Dexing Dai, Ruoman Sun, Yali Ling, Lei Qiu, Rui Wang, Ya Ding, Zhongjian Xie

**Affiliations:** National Clinical Research Center for Metabolic Diseases, Hunan Provincial Key Laboratory of Metabolic Bone Diseases, and Department of Metabolism and Endocrinology, The Second Xiangya Hospital of Central South University, Changsha, Hunan, China

**Keywords:** vitamin D analogues, falls, muscle strength, randomized controlled trials, meta-analysis

## Abstract

**Background:**

Elderly people are at high risk of falls due to decreased muscle strength. So far, there is currently no officially approved medication for treating muscle strength loss. The active vitamin D analogues are promising but inconsistent results have been reported in previous studies. The present study was to meta-analyze the effect of active vitamin D analogues on muscle strength and falls in elderly people.

**Methods:**

The protocol was registered with PROSPERO (record number: CRD42021266978). We searched two databases including PubMed and Cochrane Library up until August 2023. Risk ratio (RR) and standardized mean difference (SMD) with 95% confidence intervals (95% CI) were used to assess the effects of active vitamin D analogues on muscle strength or falls.

**Results:**

Regarding the effects of calcitriol (n= 1), alfacalcidol (n= 1) and eldecalcitol (n= 1) on falls, all included randomized controlled trials (RCT) recruited 771 participants. Regarding the effects of the effects of calcitriol (n= 4), alfacalcidol (n= 3) and eldecalcitol (n= 3) on muscle strength, all included RCTs recruited 2431 participants. The results showed that in the pooled analysis of three active vitamin D analogues, active vitamin D analogues reduced the risk of fall by 19%. Due to a lack of sufficient data, no separate subgroup analysis was conducted on the effect of each active vitamin D analogue on falls. In the pooled and separate analysis of active vitamin D analogues, no significant effects were found on global muscle, hand grip, and back extensor strength. However, a significant enhancement of quadriceps strength was observed in the pooled analysis and separate analysis of alfacalcidol and eldecalcitol. The separate subgroup analysis on the impact of calcitriol on the quadriceps strength was not performed due to the lack to sufficient data. The results of pooled and separate subgroup analysis of active vitamin D analogues with or without calcium supplementation showed that calcium supplementation did not affect the effect of vitamin D on muscle strength.

**Conclusions:**

The use of active vitamin D analogues does not improve global muscle, hand grip, and back extensor strength but improves quadriceps strength and reduces risk of falls in elderly population.

## 1 Introduction

An increased risk of falls associated with decreased muscle strength and increased bone fragility is a major concern for older adults due to high disability and mortality rates, as well as decreased quality of life. Observational studies have shown a positive correlation between decreased muscle strength and vitamin D deficiency (1–3). Vitamin D deficiency is also associated with increased risk of sarcopenia (4). Vitamin D supplementation has been recommended for maintaining and improving musculoskeletal health (5). Numerous randomized clinical controlled trials (RCTs), systematic reviews and meta-analyses have focused on native vitamin D including vitamin D_2_ (also known as ergocalciferol) and vitamin D_3_ (also known as cholecalciferol) and demonstrated the effects of vitamin D supplementation on improving muscle strength (6, 7) and reducing the risk of falls (6, 8, 9). However, other meta-analyses showed inconsistent results (10, 11), which may be due to differences in experimental design and treatment doses of included studies.

In recent clinical trials, 1α,25-dihydroxyvitamin D_3_ (also known as calcitriol) (12, 13), the physiologically active form of vitamin D, and its prodrug, 1α-hydroxyvitamin D_3_ (also known as alfacalcidol) (14), have been shown to improve hand grip strength, while some RCTs showed conflicting results (15, 16). Supplementations of alfacalcidol have been shown to enhance quadriceps muscle strength (17). However, alfacalcidol does not seem to improve back extensor strength in the older subgroup (18). As for the new vitamin D analogue, eldecalcitol, Saito, et al. (19) reported its positive effects on improving quadriceps muscle strength and back extensor strength, but not on hand grip strength. Another clinical trial showed that eldecalcitol does not increase the strength of back extensor and knee extensor after 6 months of treatment (20). Regarding the effect of active vitamin analogues on falls, it has been shown that supplementation with calcitriol (21) or alfacalcidol (22) reduces the incidence rate and cumulative number of falls in older adults. However, another study (19) has shown that eldecalcitol has no effect on reducing the number of falls.

Alfacalcidol is converted to calcitriol through hydroxylation in the liver (23). Since eldecalcitol is not a prodrug of calcitriol, it does not directly increase levels of the natural ligand (24). Compared with calcitriol, eldecalcitol has a stronger affinity for the vitamin D binding protein (DBP) and a weaker affinity for the vitamin D receptor (VDR) (25). The effects of eldecalcitol on inducing cell differentiation (26) and reducing serum parathyroid hormone (PTH) levels (27) seems to be weaker than calcitriol and alfacalcidol. RCTs have shown that eldecalcitol increases bone mineral density and reduces the incidence of vertebral fractures better than calcitriol (25) and alfacalcidol (28). Although the three types of active vitamin D analogues exhibit differences in receptor affinity, DBP binding ability, and metabolism, which may have different effects on enhancing muscle strength and preventing falls, no comparative RCTs have been conducted to evaluate the effects of calcitriol, alfacalcidol, and eldecalcitol on muscle strength and preventing falls. Although several meta-analyses (29–31) have been conducted to investigate the effects of calcitriol and alfacalcidol on falls, and the results have shown a protective effect on fall prevention, there is still no updated and more comprehensive meta-analyses to address effects of vitamin D analogues including calcitriol, alfacalcidol and eldecalcitol on falls. In addition, published meta-analyses (32, 33) on muscle strength conduced a mixed analysis of cholecalciferol, ergocalciferol, calcitriol and alfacalcidol, but did not distinguish them. The aim of the present meta-analysis was to analyze effects of active vitamin D analogues, including calcitriol, alfacalcidol and eldecalcitol, on muscle strength and falls in elderly individuals based on the data from RCTs.

## 2 Materials and methods

### 2.1 Protocol and registration

The meta-analysis followed the Preferred Reporting Items for Systematic Reviews and Meta-analyses (PRISMA) reporting guideline (34). We prespecified the methods in a protocol that was registered with the PROSPERO database (record number: CRD42021266978).

### 2.2 Search strategy

We systematically searched two databases including Pubmed and Cochrane Library up until August 2023. Two independent investigators (A. X. and H. L.) performed the search. Each title and abstract were reviewed for final inclusion into the study. The other two investigators further discussed discrepancies in study inclusion and the fifth investigator (M. L.) performed the search to reach consensus if necessary. The full search strategy and the search terms used in the analysis are presented in **Supplementary Table 1**.

### 2.3 Inclusion criteria

RCTs were included in this meta-analysis met the following criteria: (1) RCTs with any of active vitamin D analogues including calcitriol, alfacalcidol and eldecalcitol, with or without calcium over a three month follow-up period; (2) studies using falls or muscle strength as one of the primary outcome measures; (3) studies enrolling adults (ages ≥18).

### 2.4 Exclusion criteria

Exclusion criteria were as follows: (1) RCTs with cholecalciferol or ergocalciferol as a main intervention; (2) trials without a no-treatment or placebo group; (3) duration of treatment less than 3 months; (4) trials using muscle mass, muscle power, body balance or other potential end points of muscle function as outcomes; (5) trials without doses of active vitamin D analogues; and (6) conference abstracts, comments, letters, observational studies, cell or animal studies, reviews of the literature, and those without full-texts or retracted by journals.

### 2.5 Review process

Two investigators (D.D. and F.X.) conducted data extraction independently. All records were identified in the initial search and imported into EndNote X9 and duplicates were removed. The remaining records were screened for abstracts or full texts further following the inclusion and exclusion criteria. For final meta-analysis, the following information was extracted: author, year of publication, study design, sample size, characteristics of the subjects, intervention drugs (name and doses), duration of intervention, calcium doses, baseline serum 25(OH)D levels, outcome measures. Sub-group analysis was performed based on those data. For studies using falls and various types of muscle strength as outcomes or measuring the strength of the same muscle twice, for example, measuring left and right muscle strength, we extracted each outcome variable and corresponding details of each measurement based on the type of outcome variables and measurement time, as a separate entry. The extracted information was used for subsequent meta-analysis and subgroup analysis. Discrepancies in the review process were resolved by a third investigator (X.X.).

In order to include a maximum of studies in our meta-analysis, we paid particular attention to missing data. We contacted authors or coauthors when information was missing in the full-text paper. For the studies that show only medium range or mid-quartile range and the sample size, we estimated the mean and variance based on those values using the method as previously described (35, 36).

### 2.6 Quality assessment

The study quality was independently assessed by two investigators (L.L. and L.Q.). The Cochrane Collaboration risk of bias tool (37) was used to assess the quality of the followings in the selected studies: sequence generation, allocation concealment, blinding of participants, staff, and outcome assessors, completeness of outcome data, and evidence of selective outcome reporting and other potential threats to validity. According to the scoring system, study bias was defined as ‘high’, ‘some concerns’, or ‘low’. Disagreements between authors were resolved by consensus between two independent reviewers (Y.D. and R.W.).

### 2.7 Meta-analysis

Meta-analysis was performed using the Stata SE version 15.1 (STATA Corp, College Station, Tex). A random effects model was used and risk ratio (RR) and standardized mean difference (SMD) with 95% confidence intervals (95% CI) were calculated to compare falls and muscle strength outcome measures respectively. Data was divided into calcitriol, alfacalcidol and eldecalcitol groups. The variability between the studies identified determine the heterogeneity. I^2^ statistic was employed to calculate the heterogeneity of this study. I^2^ values of 25 or less, 50, and at least 75% represent low, moderate, and high inconsistency, respectively. Publication bias was assessed by using Begg’s funnel plot and Egger’s statistical tests (38).

### 2.8 Subgroup and sensitivity analyses

In order to clarify the role of whether calcium supplementation enhances the effect of active vitamin D analogues on muscle strength, we conducted subgroup analysis for vitamin D alone or in combination with calcium in pooled analysis of the three active vitamin D analogues and separate analysis of each active vitamin D. We classified some studies that did not provide information on calcium supplementation as studies without calcium supplementation. Sensitivity analyses were performed using one-study-removed analysis to determine the relative impact of each trial on the overall risk estimate.

## 3 Results

### 3.1 Study characteristics

**Figure 1:**
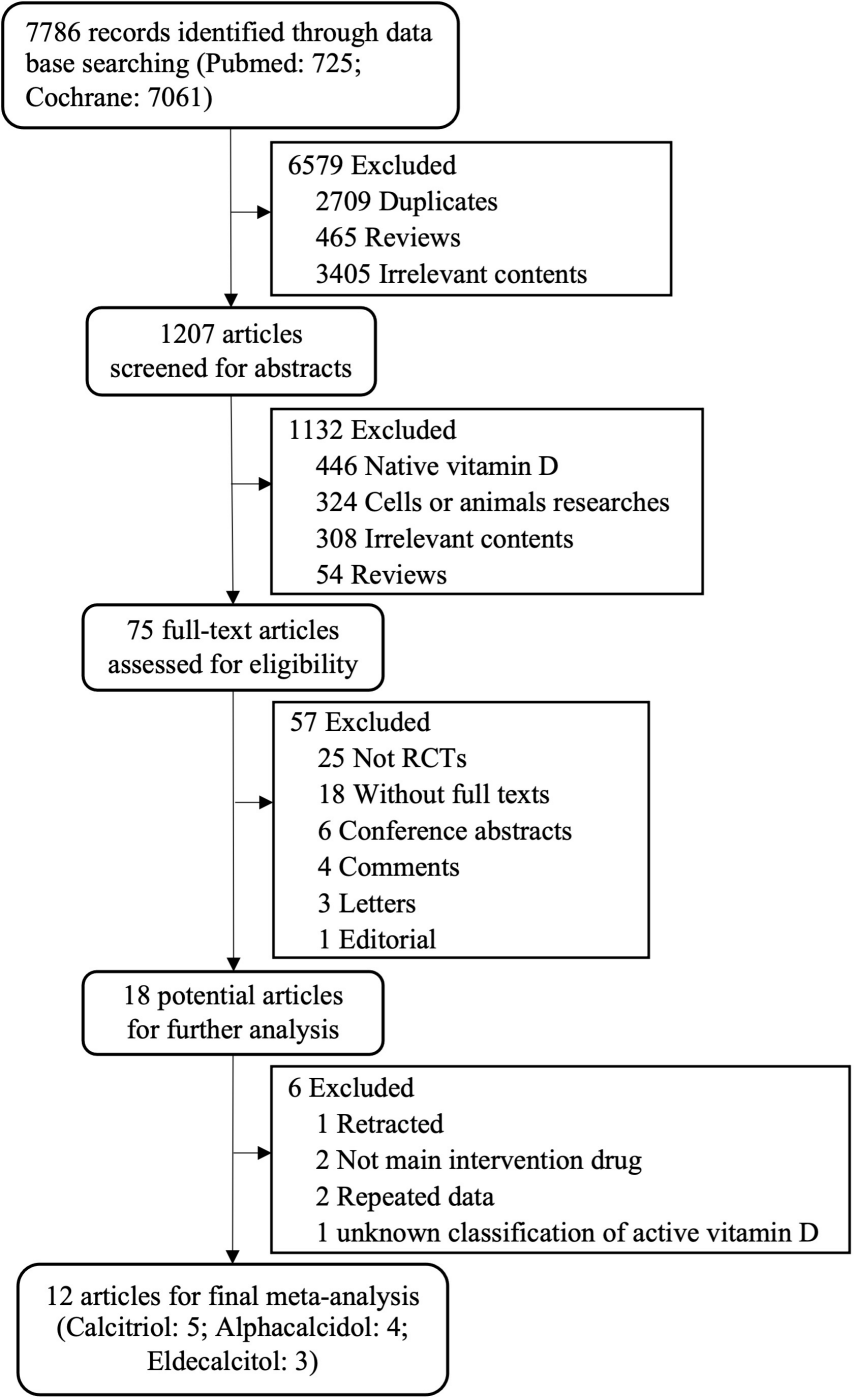
Study selection flow diagram. RCTs, randomized controlled trials.

A total of 7786 records were obtained through initial searching. There were 1207 articles screened for abstracts for further selection after removing duplicates, reviews and irrelevant contents. During the full-text review, 6 studies were identified as presenting incomplete or missing data. We contacted the authors of those studies and obtained the required data for one of the studies (13). Five of them were changed to mean and variance for further analysis according to medium, range, or mid-quartile range and the sample size (14, 15, 17, 19, 20). Finally, we identified 12 RCTs in which active vitamin D analogues (calcitriol (12, 13, 15, 16, 21): n=5, alfacalcidol (14, 17, 18, 22): n=4, eldecalcitol (19, 20, 39): n=3) were used (**Figure 1**).

The basic characteristics of the included trials were shown in **Table 1**. About 90% of the participants were females with mean age 70.4 years old. Three of the included trials reported results of falls (19, 21, 22). Ten of the included trials reported results of muscle strength (12–20, 39). One of the included trials reported results of both of falls and muscle strength (19). In the majority of the selected trials (12–17, 21, 22), participants received treatment with active vitamin D analogues alone. In four of 12 included trials, participants received treatment of vitamin D analogues combined with bisphosphonates or denosumab (18–20, 39). In 8 trials (12, 14, 16–18, 20–22), participants received treatment with calcitriol or eldecalcitol combined with calcium supplement. In 1 trial (13), participants received alfacalcidol alone. In 3 trials (15, 19, 39), it is unknown whether calcium supplementation was given or not. The daily doses for active vitamin D analogues were 0.25 or 0.5 μg/day for calcitriol, 0.5 or 1 μg/day for alfacalcidol, and 0.75 μg/day for eldecalcitol. The treatment period was 3 to 36 months.

**Table 1:** Characteristics of the included trials.

Study	Sample size (control/intervention)	Age (y)	Country/Sex	Intervention (name and daily dose)	Calcium supplement (mg/day)	Duration (months)	Baseline 25(OH)D (nmol/L) (control/intervention)	Outcome measures
Gallagher et al., 2004 (21)	112/101	66.0-77.0	USA / Females	Calcitriol 0.5 μg vs Placebo	Below 1000	36	80.5 ± 27.4 / 78.0 ± 21.6	Falls
Gao et al., 2015 (12)	262/116	63.4 ± 5.0	China / Females	Calcitriol 0.25 μg+ Calcium 600 mg vs Cholecalciferol 800 IU + Calcium 600 mg	600	24	62.5 (50.0–75.3)/64.4 (51.1–76.3)	Grip strength
Xia et al., 2009 (16)	76/74	70.4 ± 3.6	China / Females	Calcitriol 0.25 μg + Calcium 600 mg vs Calcium 600 mg	600	12	NA	Grip strength
Grady et al., 1991 (15)	48/50	79.1 (70.0–97.0)	USA / Males and females	Calcitriol 0.25 μg vs Placebo	NA	6	65.7 ± 51.4/60.4 ± 35.3	Grip strength
Cheng et al., 2018 (13)	66/75	57.7 ± 8.1	China / Females	Calcitriol 0.5 μg vs Placebo	0	4	49.3 ± 30.0/53.4 ± 24.3	Grip strength
Dukas et al., 2004 (22)	187/193	75.0	Switzerland / Males and females	Alphacalcidol 1 μg vs Placebo	Below 500	9	88.3 ± 33.4/93.3 ± 36.2	Falls
Setiati et al., 2017 (14)	48/47	70.0	Indonesia / Females	Alfacalcidol 0.5 μg vs Placebo	500	3	37.5 (17.0–66.0)/42.0 (19.0–96.0)	Grip strength
Hara et al., 2013 (18)	48/53	55.0-75.0	Japan / Females	Alfacalcidol 1 μg + Alendronate 35 mg vs Alendronate 35 mg	200	4	57.4 ± 20.3/56.8 ± 20.0	Back extensor strength
Songpatan/asilp et al., 2009 (17)	19/21	70.6 ± 4.3	Thai / Females	Alfacalcidol 0.5 μg vs Placebo	1500	3	74.3 ± 10.1/77.1 ± 14.3	Quadriceps muscle strength
Saito et al., 2021 (19)	91/89	55.0-75.0	Japan / Females	Eldecalcitol 0.75 μg + Bisphosphonate vs Bisphosphonate	NA	6	NA	Falls/Multiple muscle strength
Saito et al., 2016 (39)	17/18	74.0(63.0–86.0)	Japan / Females	Eldecalcitol 0.75 μg + Alendronate 35 mg/week vs Alendronate 35 mg/week	NA	6	NA	Multiple muscle strength
Miyakosh et al., 2020 (20)	13/8	>60.0	Japan / Females	Eldecalcitol 0.75 μg + Denosumab vs Cholecalciferol 400 IU + Denosumab	610	6	85.8 (78.0–111.7)/74.9 (52.4–113.9)	Multiple muscle strength

NA, Not Available.

### 3.2 Risk of bias of included studies

The overall quality of the included studies was good, as shown in **Supplementary Figure 1**. One trial (20) in which the strength of four types of muscle was measured as outcomes had high risk of bias due to missing outcome data. The risk of bias in deviating from scheduled interventions in two studies (12, 13) was considered “some concerns” because they may not have evaluated the effectiveness of the interventions assigned. One study (12) showed a high risk of bias arising from the randomization process due to the lack of information about concealment of treatment allocation. There were some issues of bias in reported results of three studies (16, 17, 39) as these results are likely to be selected from multiple eligible data analyses.

### 3.3 Meta-analysis of RCTs of vitamin D analogues for the prevention of falls

**Figure 2:**
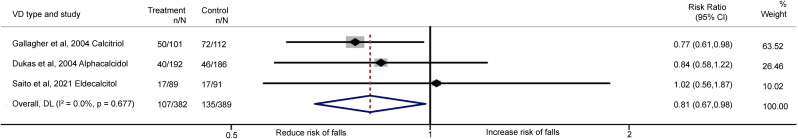
Effects of vitamin D analogue supplementation on falls.

There were 3 trials in which the number of fallers were measured as outcome variables [calcitriol (21): n= 1; alfacalcidol (22): n= 1; eldecalcitol (19): n= 1]. These studies enrolled 771 participants in total. The average age varied from 55-75 years old. As shown in **Figure 2**, the meta-analysis showed that treatment with active vitamin D analogues significantly reduced the risk of falls with a RR of 0.81 (95% CI= 0.67 to 0.98) in pooled analysis of three active vitamin D analogues. No heterogeneity was found in this meta-analysis (I^2^ = 0.00%; p= 0.677). In one-study-removed sensitivity analyses, we found that the results of the meta-analysis had no change when each trial was excluded (**Supplementary Figure 4**). Due to limited number of the studies included, we were unable to conduct separate analysis or sub-group analysis to determine whether each active vitamin D analogue had an impact on falls and whether calcium affects the action of active vitamin D analogues.

### 3.4 Meta-analysis of RCTs of vitamin D analogues and muscle strength

**Figure 3:**
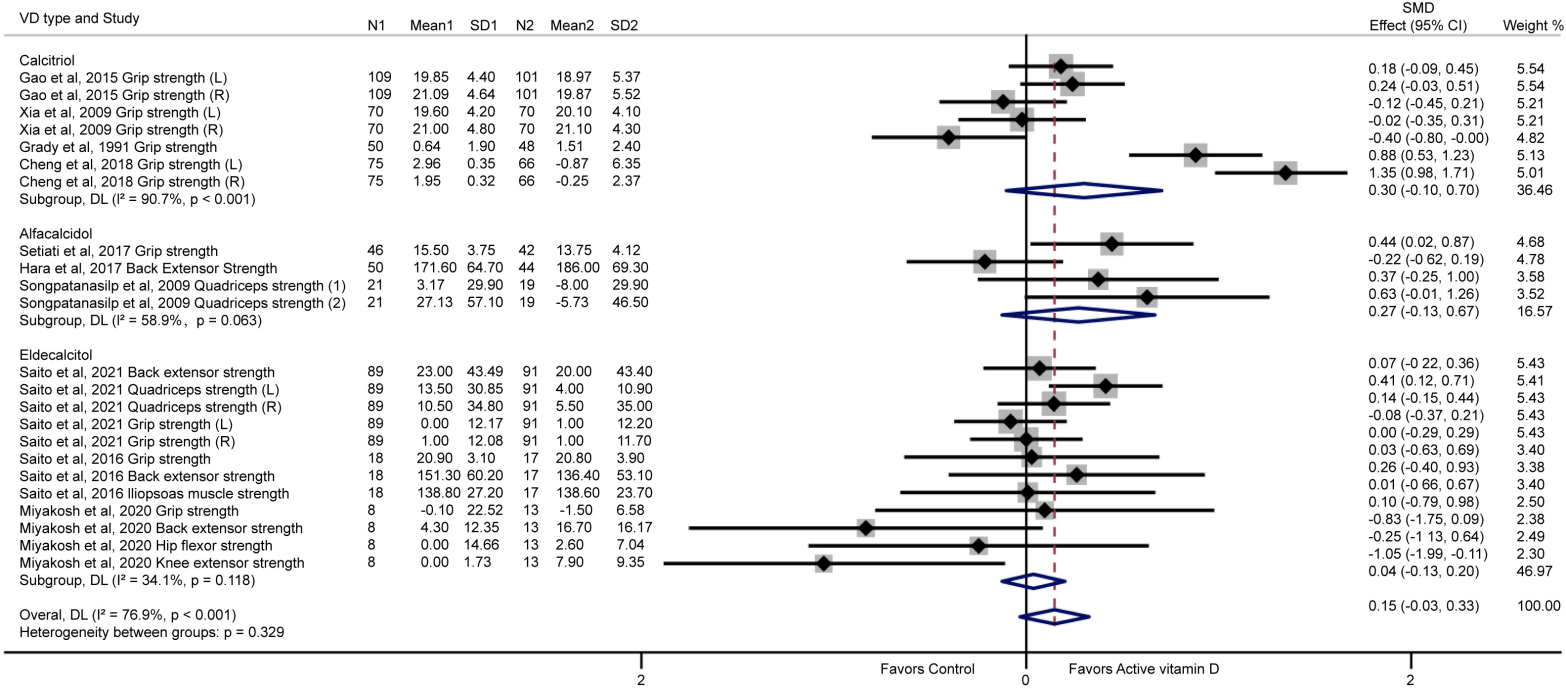
Effects of vitamin D analogue supplementation on global muscle strength. Grip strength represents hand grip strength; (L) represents left; (R) represents right; (1) represents quadriceps strength 30°/sec; (2) represents quadriceps strength 60°/sec.

Ten trials were included in the meta-analysis of vitamin D analogues and muscle strength (calcitriol (12, 13, 15, 16): n= 4; alfacalcidol (14, 17, 18): n= 3; eldecalcitol (19, 20, 39): n= 3). In 3 of the studies (19, 20, 39) muscle strength at more than two sites was measured. In 5 of the studies (12, 13, 16, 17, 19), the muscle strength of the same site was measured once on the left and right sides, or twice on the same side. In three of the studies (14, 15, 18) muscle strength was measured at a single site. Totally, 23 items containing 2431 participants was included in the final meta-analysis. The mean age of participants in each trial varied from 57.7 to 79.1 years old. The results of the meta-analysis showed that none of the active vitamin D analogues including calcitriol (SMD= 0.30; 95% CI= -0.10 to 0.70), alfacalcidol (SMD= 0.27; 95% CI= -0.13 to 0.67), and eldecalcitol (SMD= 0.04; 95% CI= -0.13 to 0.20) had an effect on global muscle strength separately (**Figure 3**). Moreover, pooled analysis of all active vitamin D analogues also showed no effect on improving global muscle strength (SMD= 0.15; 95% CI= -0.03 to 0.33) (**Figure 3**).

The heterogeneity of this pooled meta-analysis was high (I^2^ = 76.9%; p< 0.001), which may be due to the fact that multiple muscle types were tested and different types of active vitamin D analogues were used. Funnel plot analysis showed no obvious publication bias (**Supplementary Figure 2**). In addition, similar inference was noted here according to the Egger’s test (p= 0.465). The results of meta-analysis remained statistically significant when any of the individual trials was removed (**Supplementary Figure 5**).

**Figure 4:**
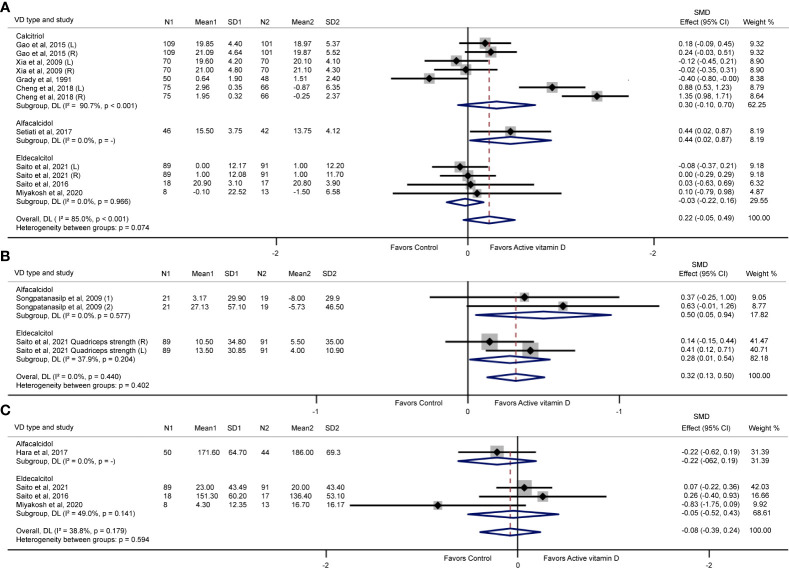
Effects of vitamin D analogue supplementation on hand grip strength **(A)**, quadriceps strength **(B)**, and back extensor strength **(C)**. (L) represents left; (R) represents right; (1) represents quadriceps strength 30°/sec; (2) represents quadriceps strength 60°/sec.

Eight trials (12–14, 16, 18–20, 39) reported results of hand grip strength. Four trials (18–20, 39) reported results of back extensor strength and two trials (17, 19) reported results of quadriceps strength. Iliopsoas muscle strength (39), hip flexor strength (20) and knee extensor strength (20) were reported once in each of three trials (**Figure 3**). We conducted a meta-analysis for the effect of the active vitamin D analogues on hand grip strength, quadriceps strength and back extensor strength (**Figure 4**). The results showed that active vitamin D analogues did not increase the hand grip strength (SMD= 0.22; 95% CI= -0.05 to 0.49) (**Figure 4A**). Separate subgroup analysis of calcitriol or eldecalcitol also did not affect the hand grip strength enhancement (SMD= -0.30; 95% CI= -0.10 to 0.70 and SMD= -0.03; 95% CI= -0.22 to 0.16). However, only a small improvement was observed in a small sample size study of alfacalcidol (SMD= 0.44; 95% CI= 0.02 to 0.87), and it is difficult to conclude alfacalcidol treatment increases hand grip strength. Interestingly, we found that treatment with active vitamin D analogues (alfacalcidol or eldecalcitol) resulted in significant improvements of quadriceps strength with a total SMD of 0.32 (95% CI= 0.13 to 0.50), a SMD of 0.50 (95% CI=0.05 to 0.94) for alfacalcidol and a SMD of 0.28 (95% CI= 0.01 to 0.54) for eldecalcitol, respectively (**Figure 4B**). **Figure 4C** shows that alfacalcidol or eldecalcitol had no effects on improving back extensor strength (SMD= -0.22; 95% CI= -0.62 to 0.19 and SMD= -0.05; 95% CI= -0.52 to 0.43). Nevertheless, there is currently a lack of study on the effects of calcitriol on quadriceps and back extensor strength.

We found high heterogeneity in the meta-analysis of hand grip strength (I^2^ = 85.0%; p< 0.001) and modest heterogeneity in the meta-analysis of back extensor strength (I^2^ = 38.8%; p= 0.179), but no heterogeneity in the meta-analysis of quadriceps strength (I^2^ = 0.0%; p= 0.440). The high heterogeneity may be caused by differences in study designs or protocols, subject populations, drug types and intervention lengths. Funnel plot analysis showed no obvious publication bias in the test of hand grip strength (**Supplementary Figure 3**). This result was in line with the results obtained with Egger’s test (hand grip strength: p= 0.846). Publication bias could not be assessed in the tests of quadriceps strength and back extensor strength because of the included the number of trials was smaller than 10. In one-study-removed sensitivity analyses, we also found no change in the meta-analysis when a trial was removed (**Supplementary Figures 6–8**).

In subgroup analyses (**Table 2**), the results showed that in all included studies, calcium supplementation did not enhance the effect of active vitamin D on improving muscle strength, with a SMD of 0.054 (95% CI = -0.14 to 0.25) for calcium supplementation and a SMD of 0.25 (95% CI = -0.04 to 0.54) for no calcium supplementation (**Table 2**). Similarly, in the subgroup analysis of calcitriol or eldecalcitol, there was no significant impact of calcium supplementation on the effect of vitamin D on improving muscle strength (**Table 2**). Due to the supplementation of calcium in all four studies on the treatment of alfacalcidol, we could not compare whether supplementing calcium or not supplementing calcium could enhance the effect of alfacalcidol on muscle strength. In the pooled and separate analysis of active vitamin D analogues, calcium supplementation did not have a significant effect on improving muscle strength.

**Table 2:** Subgroup analyses of the effects of calcium combined with active vitamin D analogues on muscle strength.

Subgroup			Studies, n	Participants, n		SMD [95% Conf. Interval]	I^2^, %	*p* values
				Treatment	Control			
Active vitamin D analogues in the pooled analysis								
		Without calcium	11	699	686	0.25 [-0.04, 0.54]	85.00	0.092
		With calcium	12	528	518	0.054 [-0.14, 0.25]	50.10	0.58
Active vitamin D analogues in the separate analysis								
**Calcitriol**							
		Without calcium	3	200	180	0.61 [0.37, 1.59]	95.30	0.22
		With calcium	4	358	342	0.093 [-0.07, 0.26]	16.00	0.26
**Edecalcitol**								
		Without calcium	8	499	506	0.11 [-0.018, 0.23]	0.00	0.093
		With calcium	4	32	52	-0.49 [-1.01, 0.025]	22.90	0.062

## 4 Discussion

### 4.1 Main findings

Out of the 1207 potentially relevant articles, 12 RCTs were included in the meta-analysis, involving 771 individuals with fall outcomes and 2431 individuals with muscle strength outcomes. The results showed that supplementation with vitamin D analogues reduced the risk of falls by 19% among elderly population. However, regardless of whether individuals received additional calcium supplementation, the effect of active vitamin D treatment on global muscle strength was not significant. Nevertheless, the effect of active vitamin analogues on enhancing muscle strength, especially on enhancing quadriceps strength was observed. It has been shown that quadriceps strength is an important predictor of incident falls (40). Therefore, the active vitamin D analogues reduce the risk of falls possibly by increasing quadriceps strength. Moreover, although there was no direct comparison of these three active vitamin D analogues, their spectrum of action in enhancing muscle strength is the same.

### 4.2 Comparison with previous meta-analyses

The reduced risk of falls in the present meta-analysis is consistent with the previous meta-analyses by Richy et al. (31) (RR = 0.79, 95% CI= 0.64–0.96), Bischoff-Ferrari et al. (29) (RR= 0.78, 95% CI= 0.64-0.94), and Siobhan et al. (30) (OR = 0.66, 95% CI= 0.44-0.98) who analyzed effects of calcitriol and alfacalcidol on the risk of falls. These studies were published in 2008 and 2009, respectively. The present meta-analysis included vitamin D analogues, such as calcitriol, alfacalcidol and eldecalcitol, as well as a larger sample sizes, providing more comprehensive and up-to-date evidence to demonstrate the effect of active vitamin D analogues on muscle strength and falls. In a previous meta-analysis on the impact of active vitamin D analogues on falls, studies on active vitamin D analogues such as calcitriol and alfacalcidol were mixed with studies on cholecalciferol for analysis (41). The present meta-analysis excluded studies using vitamin D and only included studies using active vitamin D analogues.

As for muscle strength, similarly, all available meta-analyses were conducted using a mixture of vitamin D and active vitamin D analogues. None of the meta-analyses investigated the effect of active vitamin D analogs alone on muscle strength. Rosendahl-Riise, et al. (32) enrolled 3 studies on calcitriol and alfacalcidol and 12 studies on cholecalciferol and ergocalciferol in one pooled meta-analysis in which only hand grip strength was measured, and the results showed no significant improvement in hand grip strength in community-dwelling elderly. Recently, a meta-analysis (42) of a larger number of eligible RCTs has demonstrated insignificant improvements in hand grip strength based on a mixed analysis of cholecalciferol, ergocalciferol, calcifediol, calcitriol, alfacalcidol and eldecalcitol. Similar results were found on hand grip strength and back muscle strength among postmenopausal women (43). However, a recent meta-analysis (33) showed improved hand grip strength using mixed data of cholecalciferol, ergocalciferol, calcifediol, calcitriol and alfacalcidol trials. The present meta-analysis included RCTs in which only active vitamin D analogues was used and various type of muscle strength was measured, and the results showed no significant improvements in hand grip strength and back muscle strength with active vitamin D analogues.

Another study (44), which included studies with various doses of cholecalciferol, ergocalciferol, calcitriol and alfacalcidol supplementation, has shown a small but a significant positive effect of vitamin D and active vitamin D supplementation on global muscle strength. Although we failed to detect any significant effect of active vitamin D analogues on global muscle strength, our results showed a specific increasement in quadriceps strength after the treatment of active vitamin D analogues. However, we find no effect of vitamin D analogue treatment on hand grip strength and back extensor strength. The possible reason why the active vitamin D analogues only has an effect on quadriceps muscle strength but no effects in upper limb muscle strength (hand grip strength) or trunk muscle strength (back extensor strength) may be related to following factors. Firstly, the present meta-analysis included studies of upper limb muscle and trunk muscle with negative effects increased the weighting in the pooled analyses. Secondly, VDR polymorphisms may had different effects on upper, lower limb and trunk muscles. A significant association between the VDR genotypes and quadriceps and grip strength has been observed in elderly (45). It was reported that subjects with the presence of (allele bb) phenotype of a restriction fragment had 23% stronger muscle strength in the quadriceps than those of allele BB phenotype (45). Thirdly, vitamin D deficiency is associated with a lower proportion of type II muscle fibers, also known as fast muscles (46). 1,25(OH)_2_D_3_ increases expression of the fast myosin heavy chain isoform during the differentiated phase in mouse muscle cell lines (47). The findings from the present meta-analysis provide supporting data for the role of active vitamin D in increasing type II muscle fibers. Hand grip strength is influenced by a variety of upper limb muscles including both type I and type II muscle fibers. The quadriceps muscle is mainly composed of type II muscle fibers, while back muscles contain mainly type I muscle fibers (48). Thus, these muscles may have different responses to the treatment with active vitamin D analogues. Moreover, compared to the upper limbs, lower limbs are more frequently used for load-bearing in daily life and exercise. As older women age, their upper limb activity increases and lower limb activity decreases. This is because their inter-limb pattern changes during physical activity change, resulting in a more severe decrease in lower limb muscle strength (49). Quadriceps muscle is one of the important lower limb muscles related to falls (40). Therefore, the response of quadriceps to active vitamin D therapy may be better than that of upper limb and trunk muscles.

In subgroup analyses, although the combination of vitamin D and calcium appears to be more effective in treating musculoskeletal disease as previously reported (50), there was no significant difference in the effect between the active vitamin D analogues combined with calcium and the active vitamin D analogues alone in the present studies, suggesting that calcium does not seem to contribute to improving muscle strength. To determine whether the combination of anti-osteoporosis drugs and active vitamin D analogues would affect the effect of active vitamin D analogues on muscle strength, we conducted a subgroup analysis. In both pooled and separate analyses, the results showed there was no significant difference in the effectiveness of using active vitamin D analogues alone compared to using a combination therapy of bisphosphonates or denosumab and the active vitamin D analogue on improving muscle strength (see **Supplementary Table 2**). However, we were unable to perform subgroup analyses in risk of falls, as only one included study was a combination therapy study using eldecalcitol and bisphosphonates. Moreover, bisphosphonates were also used in the control group of this study, so the use of bisphosphonates had a small effect on the effectiveness of eldecalcitol in preventing falls.

An increased risk of falls is a consequence of decreased muscle function (51). Muscle function can be evaluated by muscle strength, muscle mass, and physical performance tests. Charlotte, et al. (44) reported that treatment with cholecalciferol or ergocalciferol had no significant effect on muscle mass. A mixed meta-analysis (43) also showed that treatment with cholecalciferol, ergocalciferol or active vitamin D did not affect muscle function. However, a significant increase in the timed-up-and-go test was observed with cholecalciferol or ergocalciferol supplementation in another meta-analysis (32). Nevertheless, the sample size and number of included trials in these meta-analyses were small. There is still a lack of meta-analyses about active vitamin D analogues and muscle mass and physical performance tests. The relationship between active vitamin D and muscle mass or physical performance remains unclear. The present meta-analysis only analyzed the effect of active vitamin D analogues on muscle strength without analyzing the effect of active vitamin D analogues on muscle mass and physical performance, as the number of RCTs conducted on active vitamin D analogues and muscle mass and physical performance is limited. Further studies are required to investigate the effect of vitamin D analogues on muscle mass and physical performance.

### 4.3 Limitations and strengths

There are several limitations in the present study. First, there is a significant heterogeneity in the meta-analysis on global muscle strength. This is probably caused by the large number of studies included in the meta-analysis and by the variability observed among the different protocols of treatment. However, we have used a random effect model in our meta-analyze and investigated this heterogeneity by conducting sensitivity analysis to estimate whether the results could have been affected by a single study. Second, subgroup analyses on falls were not performed and the power of this sensitivity analysis is limited, mainly due to a small number of trials included. More well-designed RCTs are needed to illustrate the effects of vitamin D analogues on falls. Third, there is no RCT directly comparing the effects of active vitamin D analogues with cholecalciferol or ergocalciferol on muscle strength and fall risk. Therefore, we have not been able to compare the advantages and disadvantages, such as efficacy and safety, of active vitamin D analogues with cholecalciferol or ergocalciferol in improving muscle strength and preventing falls. Further RCTs are needed to compare the effects of active vitamin D with cholecalciferol or ergocalciferol on muscle strength and fall risk. Moreover, the common side effects of active vitamin D analogues are hypercalcemia and hypercalciuria. However, due to the incomplete data of side effects of active vitamin D analogues in the studies included, we were unable to do a meta-analysis on the safety of active vitamin D analogues. Last, because the vast majority patients were women, we did not conduct sub-analysis of muscle strength by gender. Future trials are expected to pay more attention to the gender differences.

Our study also has some strength. In the present study, we conducted a meta-analysis to evaluate the effects of calcitriol, alfacalcidol and eldecalcitol on muscle strength and the risk of falls and the effects of active vitamin D analogues on muscle strength of several major muscle groups in the body. The results suggest that supplementation with these active vitamin D analogues help improve quadriceps strength and reduce the risk of falls in the elderly population. There was no significant difference in the spectrum of action of calcitriol, alfacalcidol and eldecalcitol in improving muscle strength. In addition, the present study also included subgroup analysis comparing the effects of using active vitamin D alone and in combination with calcium. The results indicate that when calcium supplementation is used in combination with active vitamin D analogues, they do not enhance the effect of active vitamin D analogues on improving muscle strength.

### 4.4 Summary

Administration with active vitamin D analogs in older adults is effective in reducing the risk of falls and improving quadriceps strength. However, the evidence was based on a limited number of studies and participants. Large-scale clinical trials are needed to confirm these results.

## Data availability statement

The original contributions presented in the study are included in the article/**Supplementary Material**. Further inquiries can be directed to the corresponding authors.

## Ethics statement

Ethical approval was not required for the study involving humans in accordance with the local legislation and institutional requirements. Written informed consent to participate in this study was not required from the participants or the participants’ legal guardians/next of kin in accordance with the national legislation and the institutional requirements.

## Author contributions

AX: Conceptualization, Formal analysis, Methodology, Writing – original draft. HL: Methodology, Writing – review & editing. ML: Methodology, Writing – review & editing. FX: Methodology, Writing – review & editing. XX: Methodology, Writing – review & editing. DD: Methodology, Writing – review & editing. RS: Methodology, Writing – review & editing. YL: Methodology, Writing – review & editing. LQ: Methodology, Writing – review & editing. RW: Methodology, Writing – review & editing. YD: Methodology, Writing – review & editing. ZX: Conceptualization, Funding acquisition, Project administration, Supervision, Writing – review & editing.
