# Supplementary material for: Effects of active vitamin D analogues on muscle strength and falls in elderly people: an updated meta-analysis
Source: Front Endocrinol (Lausanne). 2024 Feb 1;15:1327623. doi: 10.3389/fendo.2024.1327623 (PMC10867111; doi:10.3389/fendo.2024.1327623)
Supplement: Supplementary file 1 [file DataSheet_1.docx]

Supplementary Material

**Effects of active vitamin D analogues on muscle strength and falls in elderly: an updated meta-analysis**

**An Xiong, Haibo Li, Miaoying Lin, Feng Xu, Xuedi Xia, Dexing Dai, Ruoman Sun, Yali Ling, Lei Qiu, Rui Wang, Ya Ding, Zhongjian Xie^*^**

*** Correspondence:** Zhongjian Xie, M.D., Ph.D., E-mail: [zhongjian.xie@csu.edu.cn](mailto:zhongjian.xie@csu.edu.cn);

# Supplementary Figures and Tables

## Supplementary Figures


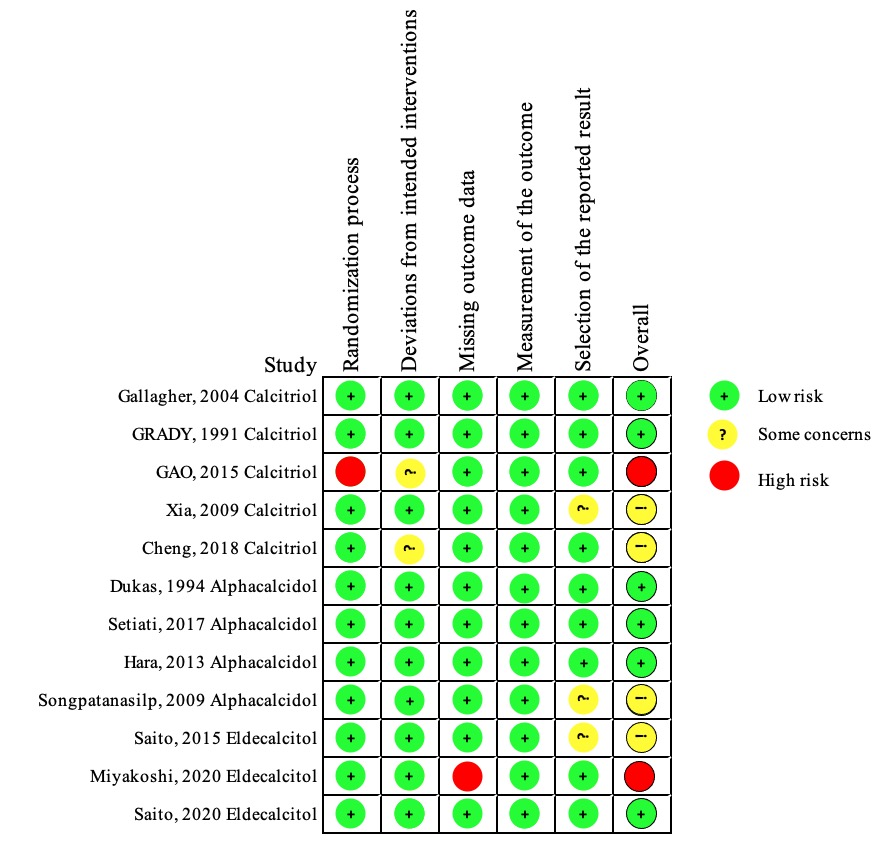


**Supplementary Figure 1.** The quality assessments using Cochrane risk of bias tool**.**


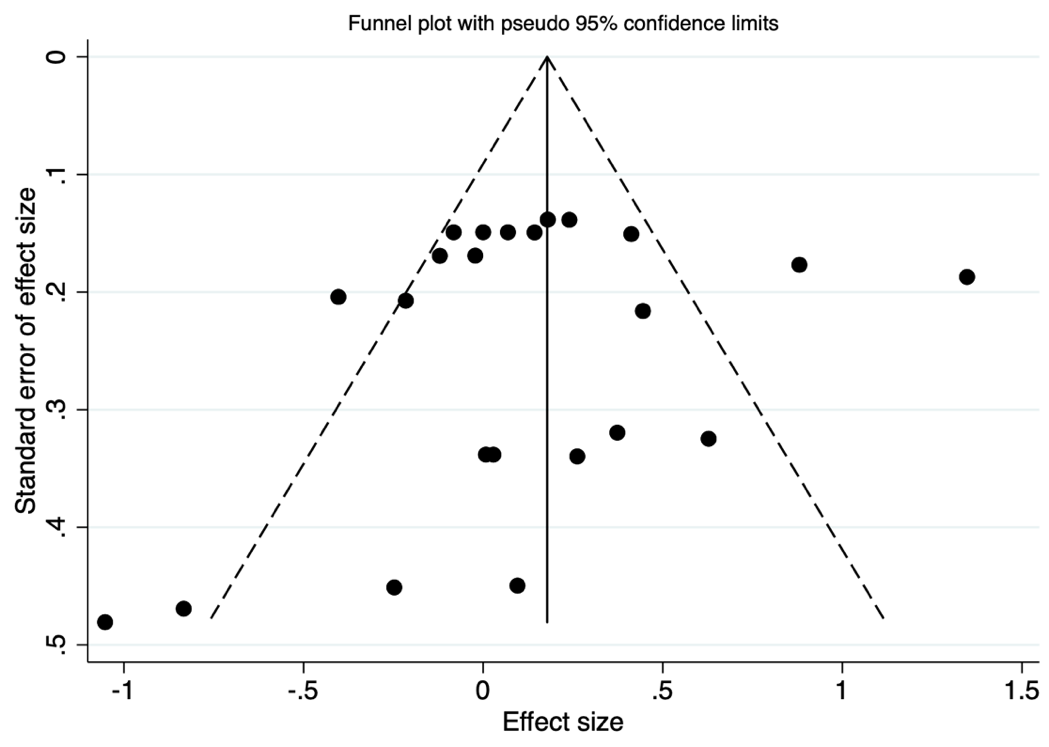


**Supplementary Figure 2.** Funnel plot analysis of effects of active vitamin D analogues on global muscle strength.


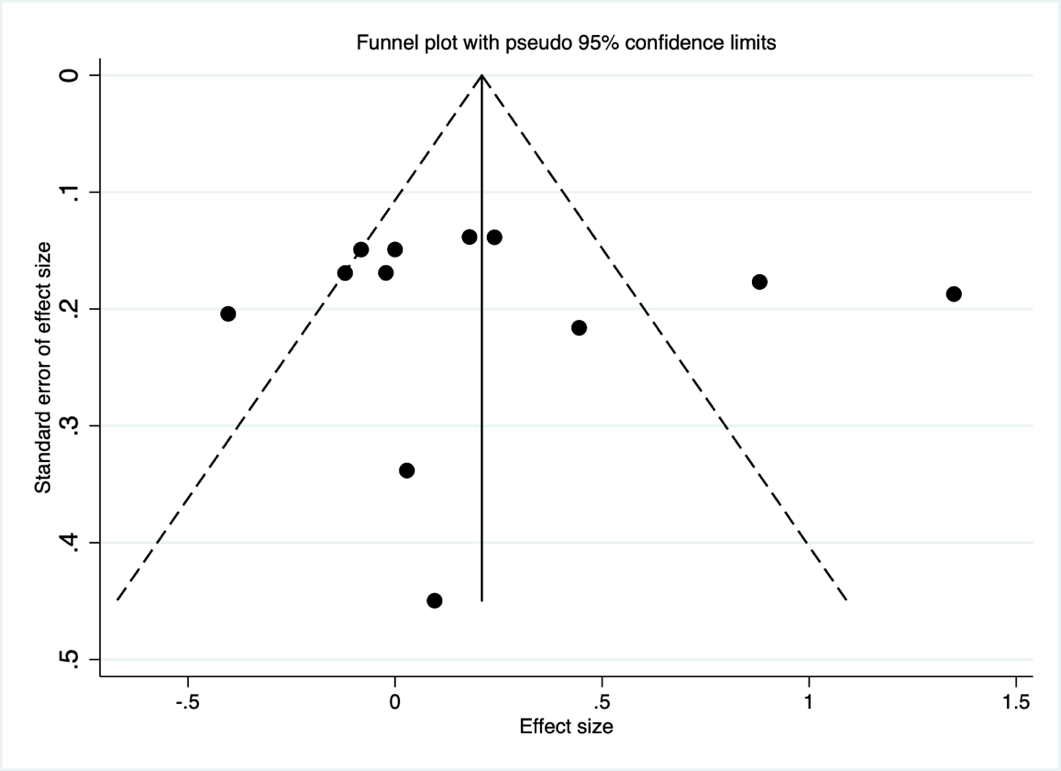


**Supplementary Figure 3.** Funnel plot analysis of effects of active vitamin D analogues on hand grip strength.


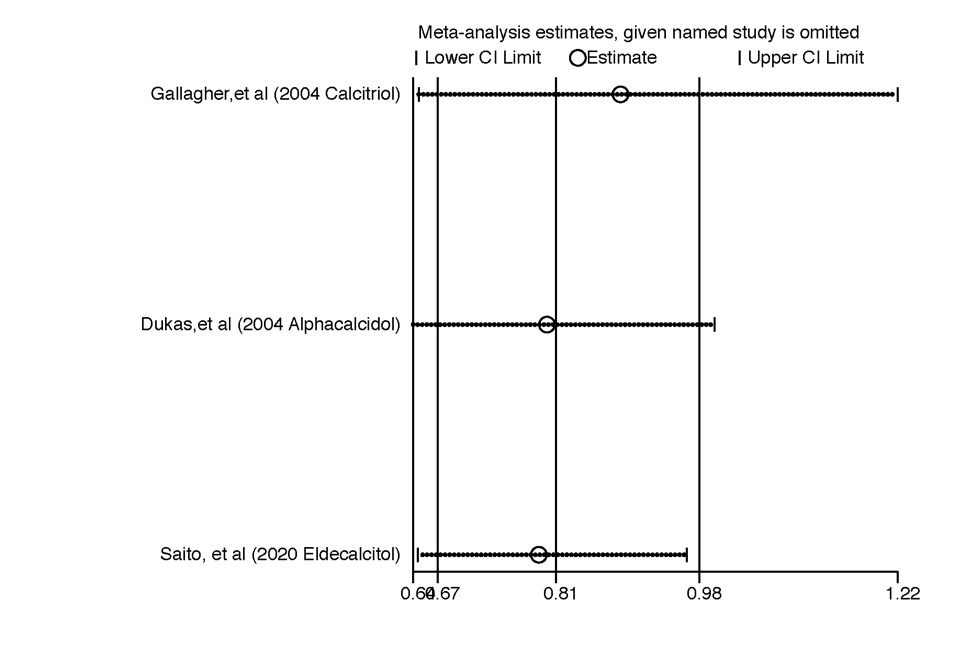


**Supplementary Figure 4.** One-study-removed sensitivity analyses of the effects of active vitamin D analogues on falls.


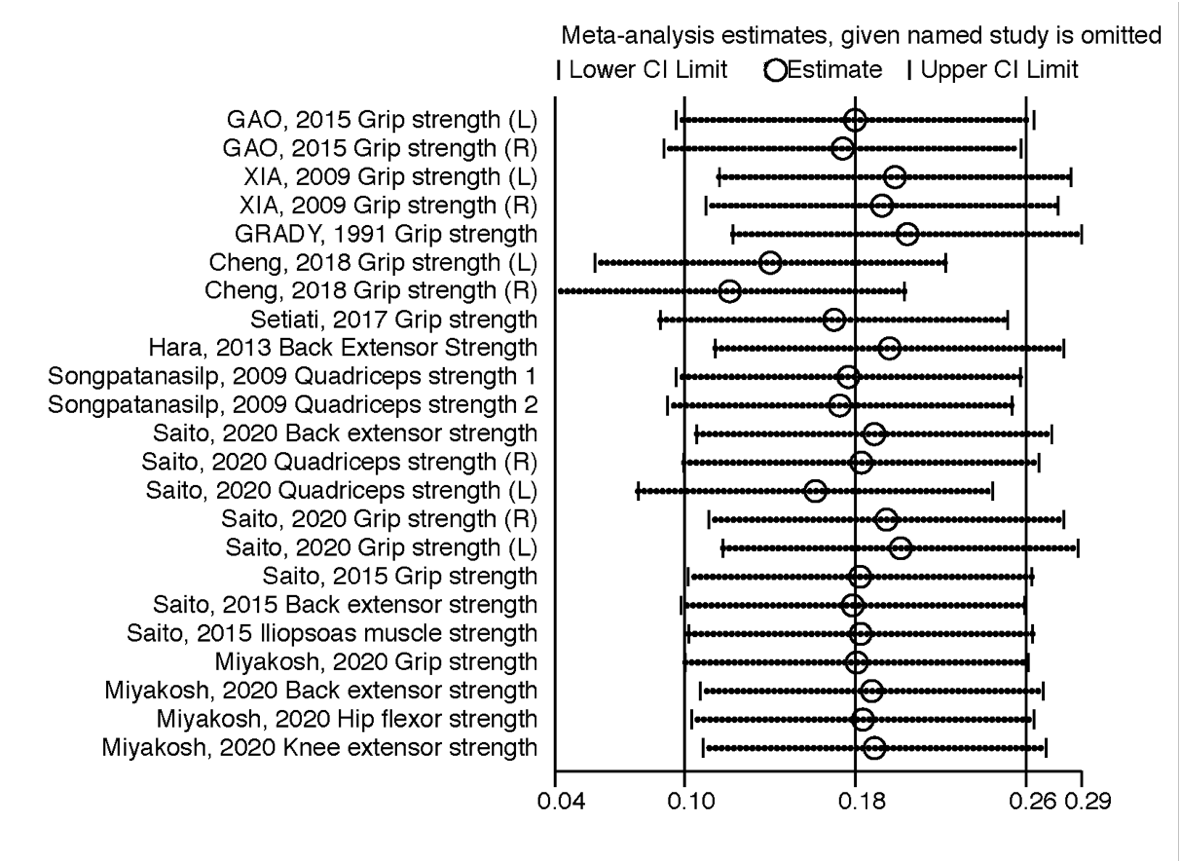


**Supplementary Figure 5.** One-study-removed sensitivity analyses of the effects of active vitamin D analogues on global muscle strength.


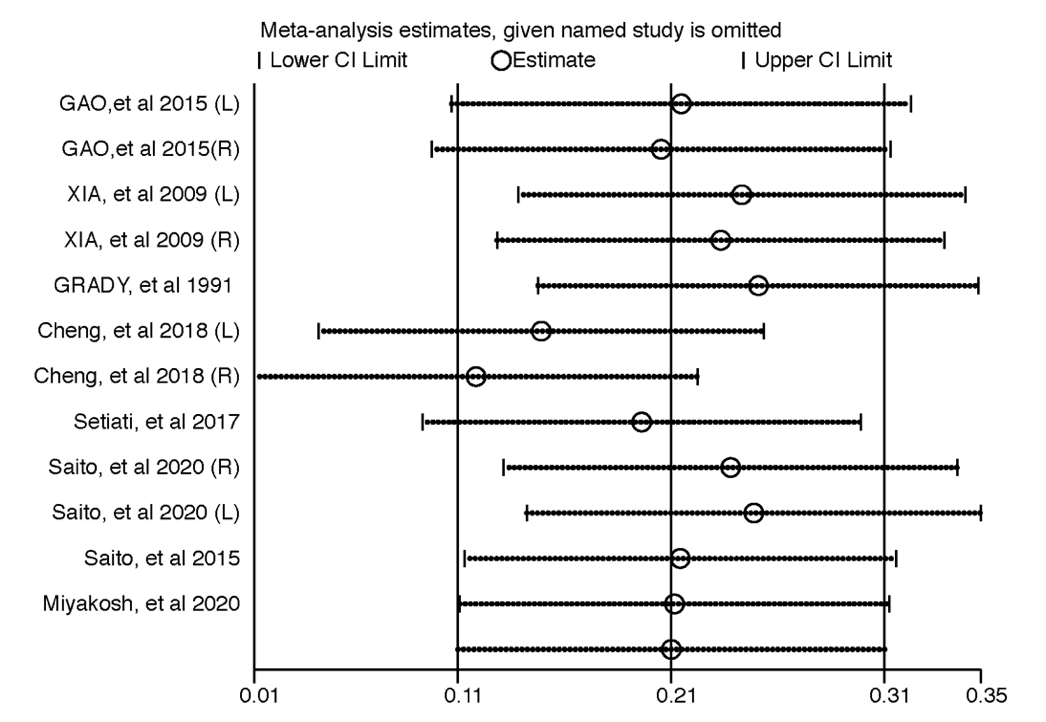


**Supplementary Figure 6.** One-study-removed sensitivity analyses of the effects of active vitamin D analogues on hand grip strength.


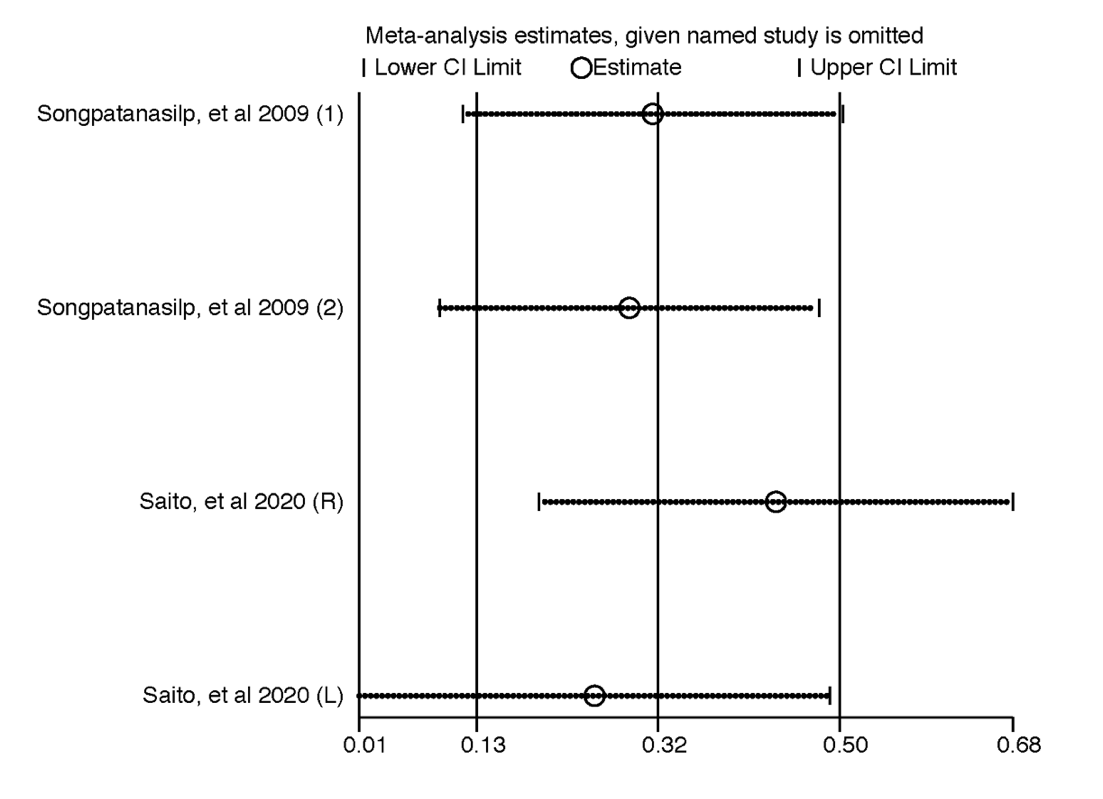


**Supplementary Figure 7.** One-study-removed sensitivity analyses of the effects of active vitamin D analogues on quadriceps strength.


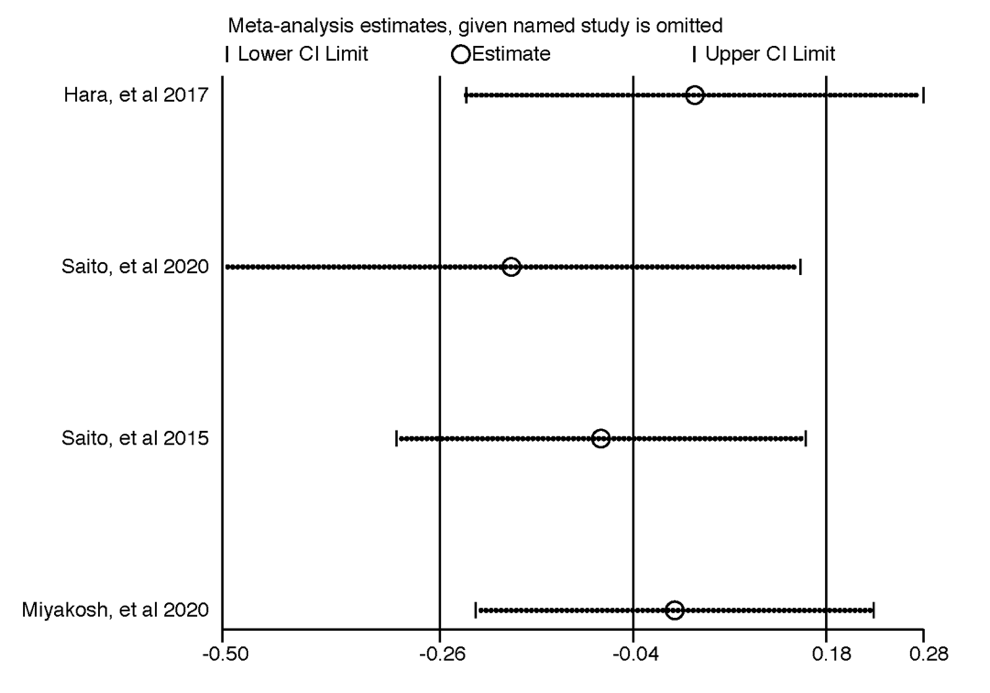


**Supplementary Figure 8.** One-study-removed sensitivity analyses of the effects of active vitamin D analogues on back extensor strength.

## Supplementary Tables

**Supplementary Table 1.** Search Strategies.

| **Database** | **Search strategy** |
| --- | --- |
| Pubmed | #1 "active vitamin D"[All Fields] OR "vitamin D analogs"[All Fields] OR "active vitamin D analogues"[All Fields] |
|  | #2 "calcitriol"[MeSH Terms] OR "calcitriol"[All Fields] OR "1,25-dihydroxyvitamin D_3_"[MeSH Terms] OR "1,25-dihydroxyvitamin D_3_"[All Fields] OR "1,25(OH)_2_D_3_"[All Fields] |
|  | #3 "alphacalcidol"[All Fields] OR "1-alpha hydroxyvitamin D_3_"[All Fields] OR "1-alpha hydroxycalciferol"[All Fields] |
|  | #4 "eldecalcitol"[All Fields] OR "2-(3-hydroxypropoxy) calcitriol"[MeSH Terms] OR "2-(3-hydroxypropoxy) calcitriol"[All Fields] OR "2-(3-hydroxypropoxy)-1,25-dihydroxyvitamin D3"[MeSH Terms] OR "2-(3-hydroxypropoxy)-1,25-dihydroxyvitamin D3"[All Fields] OR "ED-71"[All Fields] |
|  | #5 "falls"[MeSH Terms] OR "falls"[All Fields] OR "falling"[MeSH Terms] OR "falling"[All Fields] OR "accidental falls"[MeSH Terms] OR "accidental falls"[All Fields] |
|  | #6 "muscle strength"[MeSH Terms] OR "muscle strength"[All Fields] OR "muscle function"[MeSH Terms] OR "muscle function"[ All Fields] OR "muscle mass"[MeSH Terms] OR "muscle mass"[ All Fields] |
|  | #7 #1 or #2 or #3 or #4 |
|  | #8 #5 or #6 |
|  | #9 #7 and #8 |
| Cochrane library | #1 'active vitamin d': ti,ab,kw (Word variations have been searched)  #2 'vitamin D analogs': ti,ab,kw (Word variations have been searched)  #3 calcitriol: MeSH descriptor (Explode all trees) |
|  | #4 calcitriol: ti,ab,kw (Word variations have been searched) |
|  | #5 alphacalcidol: ti,ab,kw (Word variations have been searched) |
|  | #6 eldecalcitol: ti,ab,kw (Word variations have been searched) |
|  | #7 falls:ti, ab,kw (Word variations have been searched)  #8 falls: MeSH descriptor (Explode all trees) |
|  | #9 'muscle strength': ti,ab,kw (Word variations have been searched)  #10 'muscle strength': MeSH descriptor (Explode all trees) |
|  | #11 #1 or #2 or #3 or #4 or #5 or #6 |
|  | #12 #7 or #8 or #9 or #10 |
|  | #13 #11 and #12 (restricted as Cochrane Reviews or other reviews) |

**Supplementary Table 2.** Subgroup analyses of the effects of anti-osteoporotic drugs combined with active vitamin D analogues on muscle strength.

| **Subgroups** | | | | | **Studies, n** | **Participants, n** | | **SMD**  **[95% Conf. Interval]** | **I2, %** | ***p* values** |
| --- | --- | --- | --- | --- | --- | --- | --- | --- | --- | --- |
|  |  |  |  |  |  | **Intervention** | **Control** |  |  |  |
| **Active vitamin D analogues in the pooled analysis** | | |  |  |  |  |  |  |  |  |
|  |  | Without anti-osteoporotic drugs | | | 10 | 646 | 602 | 0.06[-0.05, 0.17] | 1.90 | 0.03 |
|  |  | With bisphosphonate | | | 9 | 549 | 550 | 0.03[-0.09, 0.15] | 0.00 | 0.60 |
|  |  | With denosumab | | | 4 | 32 | 52 | -0.41[-0.85, 0.04] | 0.00 | 0.08 |
| **Active vitamin D analogues in the separate analysis** | | |  |  |  |  |  |  |  |  |
|  | **Alfacalcidol** |  | | |  |  |  |  |  |  |
|  |  | Without anti-osteoporotic drugs | | | 3 | 88 | 80 | 0.44[-0.08, 0.95] | 60.90 | 0.10 |
|  |  | With bisphosphonate | | | 1 | 50 | 44 | 0.093 [-0.07, 0.26] | 71.20 | 0.23 |
|  | **Edecalcitol** |  | | |  |  |  |  |  |  |
|  |  | With bisphosphonate | | | 8 | 499 | 506 | 0.11 [-0.018, 0.23] | 0.00 | 0.093 |
|  |  | With denosumab | | | 4 | 32 | 52 | -0.49 [-1.01, 0.025] | 22.90 | 0.062 |
